# Similar reductions in the risk of human colon cancer by selective and nonselective cyclooxygenase-2 (COX-2) inhibitors

**DOI:** 10.1186/1471-2407-8-237

**Published:** 2008-08-14

**Authors:** Randall E Harris, Joanne Beebe-Donk, Galal A Alshafie

**Affiliations:** 1 The Ohio State University College of Medicine and Public Health, 320 West 10^th ^Avenue, Columbus, Ohio, 43210-1240, USA

## Abstract

**Background:**

Epidemiologic and laboratory investigations suggest that aspirin and other nonsteroidal anti-inflammatory drugs (NSAIDs) have chemopreventive effects against colon cancer perhaps due at least in part to their activity against cyclooxygenase-2 (COX-2), the rate-limiting enzyme of the prostaglandin cascade.

**Methods:**

We conducted a case control study of colon cancer designed to compare effects of selective and non-selective COX-2 inhibitors. A total of 326 incident colon cancer patients were ascertained from the James Cancer Hospital, Columbus, Ohio, during 2003–2004 and compared with 652 controls with no history of cancer and matched to the cases at a 2:1 ratio on age, race, and county of residence. Data on the past and current use of prescription and over the counter medications and colon cancer risk factors were ascertained using a standardized risk factor questionnaire. Effects of COX-2 inhibiting agents were quantified by calculating odds ratios (OR) and 95% confidence intervals.

**Results:**

Results showed significant risk reductions for selective COX-2 inhibitors (OR = 0.31, 95% CI = 0.16–0.57), regular aspirin (OR = 0.33, 95% CI = 0.20–0.56), and ibuprofen or naproxen (0.28, 95% CI = 0.15–0.54). Acetaminophen, a compound with negligible COX-2 activity and low dose aspirin (81 mg) produced no significant change in the risk of colon cancer.

**Conclusion:**

These results suggest that both non-selective and selective COX-2 inhibitors produce significant reductions in the risk of colon cancer, underscoring their strong potential for colon cancer chemoprevention.

## Background

Among American men and women, colon cancer is the third most frequently diagnosed malignancy and third leading cause of cancer death [1]. In the past two decades, incidence and mortality rates for colon cancer have declined by more than 20% in women and men [1,2]. While some authors attribute these downward trends to early detection and more effective therapy [2], the exact reasons are not yet fully understood. One factor that may have contributed to these declines is the widespread intake of aspirin, ibuprofen and other nonsteroidal anti-inflammatory drugs (NSAIDs)[3]. Among 22 published epidemiologic studies that focused on the association between intake of NSAIDs and the risk of human colon cancer, 20 reported statistically significant risk reductions. Meta-analysis of these data suggests that regular NSAID intake (primarily aspirin) reduces the risk of colon cancer by about 60% [4].

Two selective COX-2 inhibitors, celecoxib (Celebrex) and rofecoxib (Vioxx), were approved for the treatment of arthritis by the United States Food and Drug Administration (FDA) in 1999 [3]. Until the recall of Vioxx in September, 2004, these two compounds plus other selective COX-2 inhibitors valdecoxib (Bextra) and meloxicam (Mobic) were widely utilized in the United States for pain relief and treatment of osteoarthritis and rheumatoid arthritis [5,6]. The time period between approval of celecoxib to the recall of rofecoxib provides an approximate six-year window for evaluation of exposure to these compounds by a case control approach. The current case control study was designed to test and compare the chemopreventive value of selective and nonselective COX-2 inhibitors against human colon cancer.

## Methods

We studied 326 cases of invasive colon cancer with histological verification based upon review of the pathology records, and 652 group-matched controls with no personal history of cancer and no current gastrointestinal disease. Cases were sequentially ascertained for interview at the time of their diagnosis during 2003 through September, 2004 at The Arthur G. James Cancer Hospital and Richard J. Solove Research Institute (CHRI), Columbus, Ohio. There were no refusals to participate among cases. The controls were ascertained from the mammography unit and prostate screening services of the cancer hospital during the same time period and frequency matched to the cases at a rate of 2:1 by five-year age interval, race, and place (county) of residence. We interviewed randomly selected controls from these screening facilities throughout the time frame of the study to achieve a 2:1 ratio by gender, age, race and county of residence. Among men and women approached and eligible for participation, 95% completed the questionnaire. The protocol was approved by the Human Subjects Cancer Internal Review Board of The Ohio State University Medical Center and informed consent documentation was obtained for participants.

Critical information on exposure to NSAIDs and other factors were obtained utilizing a standardized risk factor questionnaire. The questionnaires were administered in person by trained medical personnel (who were blinded as to the purpose of the study) prior to definitive surgery or treatment for the cases and at the time of screening mammography or screening for prostate cancer for controls. The data variables collected consisted of demographic characteristics, height, weight, menstrual and pregnancy history for women, family history of colon cancer, comprehensive information on cigarette smoking, alcohol intake, pre-existing medical conditions (arthritis, chronic headache, cardiovascular conditions including hypertension, angina, ischemic attacks, stroke, and myocardial infarction, lung disease, and diabetes mellitus), and medication history including over the counter (OTC) and prescription NSAIDs, and exogenous hormones. Regarding selective COX-2 inhibitors and other NSAIDs, the use pattern (frequency, dose, and duration), and the type, (celecoxib, valdecoxib, rofecoxib, meloxicam, aspirin, ibuprofen, naproxen, indomethacin) were recorded. Data on the related analgesic, acetaminophen were collected for comparison with selective COX-2 inhibitors and other NSAIDs.

Case-control differences in means and frequencies were checked for statistical significance by t-tests and chi square tests, respectively. Effects of the selective COX-2 inhibitors as a group were quantified by estimating odds ratios and their 95% confidence intervals. Odds ratios were adjusted for age and colon cancer risk factors (family history, body mass, chronic smoking, and regular alcohol intake) by logistic regression analysis [7,8]. Adjusted estimates were obtained for specific types of compounds, e.g., aspirin, ibuprofen or naproxen, selective COX-2 inhibitors (rofecoxib and celecoxib), and acetaminophen. Estimates for selective COX-2 inhibitors were also adjusted for prior intake of other types of NSAIDs.

## Results

Pertinent characteristics of the cases and controls are given in Table 1. The cases exhibited higher frequencies of hypertension (OR = 2.87, 95% CI = 2.10–3.92) family history of colon cancer (OR = 1.58, 95% CI = 1.08–2.30) and chronic cigarette smoking (OR = 2.07, 95% CI = 1.49–2.87). Cases and controls had similar distributions of matching variables, age, gender, race and county of residence as well as education, body mass and alcohol consumption.

**Table 1:** Characteristics of colon cancer cases and controls.

**Characteristic ^a^**	**Cases (N = 326)**	**Controls (N = 652)**
Gender		
Female	45%	45%
Age (yrs)		
<50	15%	11%
50–59	22	25
60–69	32	34
>65	31	30
Mean (SEM)	63.2 (0.7)	63.5 (0.6)
Race		
Caucasian	90%	86%
Residence		
Franklin County, Ohio	81%	83%
Adjacent Counties	18	15
Other	1	2
Education		
< 12 yrs	12%	12%
12 yrs	53	55
> 12 yrs	35	33
Family History		
Positive	20%	13% (p < 0.01)
Body Mass		
BMI < 22	14%	12%
BMI 22–27	32	40
BMI > 27	55	48
Mean (SEM)	29.1 (0.5)	28.1 (0.3)
Smoking		
(>10 Pack-years)	33%	22% (p < 0.01)
Alcohol Intake		
None	47%	45%
1–2 drinks per week	36	35
> 2 drinks per week	17	20
Hypertension	47%	28% (p < 0.01)

^a ^Family History: colon cancer among first or second degree relatives; Body Mass Index = weight (kg)/ht ^2 ^(m).

Table 2 shows the comparative frequencies of the medications under study with multivariate-adjusted odds ratios and 95% confidence intervals. A significant reduction in the risk of colon cancer was observed for daily intake of selective COX-2 inhibitors for one year or more (Adjusted OR = 0.31, 95% CI = 0.16–0.57). Joint use of COX-2 inhibitors with aspirin or other NSAIDs was reported by 9.4% of subjects; however, the odds ratio for COX-2 inhibitors was not appreciably changed with additional adjustment for the prior intake of such compounds (OR = 0.40, 95% CI = 0.25–0.82). Estimates for smokers and nonsmokers and subjects with and without hypertension were also similar. When the data were stratified by gender, the risk reduction for the selective COX-2 inhibitors was stronger for women (OR = 0.20, 95% CI = 0.08–0.46) than men (OR = 0.75, 95% CI = 0.29–2.20).

**Table 2:** Odds ratios with 95% confidence intervals for colon cancer and selective cyclooxygenase-2 (COX-2) inhibitors, and over the counter nonsteroidal anti-inflammatory drugs (OTC NSAIDS).

**Compound**	**Number of Cases (%)**	**Number of Controls (%)**	**Multivariate OR^***d ***^(95% CI)**
**None/Infrequent Use**^***a***^	236 (72)	352 (54)	1.00
**COX-2 Inhibitors**^***b***^	15 (5)	53 (8)	0.31 (0.16–0.57)
**OTC NSAIDs**^***c***^			
**Aspirin**	22 (7)	88 (13)	0.33 (0.20–0.56)
**Ibuprofen/Naproxen**	13 (4)	68 (11)	0.28 (0.15–0.54)
**Acetaminophen**	12 (3)	22 (3)	0.81 (0.35–1.61)
**Baby Aspirin**	28 (9)	69 (11)	0.58 (0.35–1.02)

^a ^No use of any NSAID or analgesic or infrequent use of no more than one pill per week for less than one year;

^b ^COX-2 inhibitors include celecoxib, rofecoxib, valdecoxib, and meloxicam used daily for one year or more.

^c ^Over the counter (OTC) NSAIDs/analgesics used at least once per week for more than one year.

^d ^Multivariate odds ratios are adjusted for continuous variables (body mass) and categorical variables (hypertension, family history, smoking, and alcohol intake).

Significant risk reductions were also observed for the intake of one or more pills per week of regular aspirin (OR = 0.33, 95% CI = 0.20–0.56), and ibuprofen or naproxen (0.28, 95% CI = 0.15–0.54). Low dose (81 mg) aspirin produced a risk reduction with marginal significance (OR = 0.58, 95% CI = 0.35–1.20) whereas acetaminophen had no effect on the relative risk of colon cancer. Aspirin was used for cardioprotection by 9% of subjects taking 325 mg tablets compared to 93% of subjects taking 81 mg tablets.

Table 3 presents risk estimates for individual selective COX-2 inhibitors (celecoxib and rofecoxib) plus dose-response data for aspirin and ibuprofen. Daily use of either 200 mg celecoxib or 25 mg rofecoxib for more than one year produced similar risk reductions (65% and 68%, respectively). The average duration of use was 3.6 years. The trend data for OTC compounds suggests that 325 mg aspirin or 200 mg ibuprofen produced significant risk reductions when taken daily for 5 or more years.

**Table 3:** Odds ratios for colon cancer by dose, frequency, and duration of exposure to celecoxib, rofecoxib, aspirin, and ibuprofen.

**Compound**^***a***^	**Dose**	**Cases**	**Controls**	**Frequency of Use**	**Multivariate OR^***b ***^(95% CI)**
		**N (%)**			
**None**	0	236 (72)	352 (54)	None	
**Celecoxib**	200 mg	8 (2)	27 (4)	Daily	0.35 (0.18–0.93)
**Rofecoxib**	25 mg	7 (2) 26 (4)		Daily	0.32 (0.12–0.83)
**Aspirin**	325 mg	5 (2)	14 (2)	1–2 weekly	0.87 (0.24–3.17)
		4 (1)	8 (1)	3–6 weekly	0.73 (0.20–2.67)
		13 (4)	66 (10)	Daily	0.22 (0.12–0.41)
					*trend (p < 0.05)*
**Ibuprofen**	200 mg	7 (2)	17 (3)	1–2 weekly	0.67 (0.26–1.69)
		2 (1)	12 (2)	3–6 weekly	0.18 (0.02–1.50)
		4 (1)	39 (6)	Daily	0.19 (0.07–0.49)
					*trend (p < 0.01)*

^a ^Minimum duration of exposure: one year for celecoxib or rofecoxib, 5 years for aspirin or ibuprofen.

^b ^Multivariate odds ratios are adjusted for continuous variables (body mass) and categorical variables (hypertension, family history, smoking, and alcohol intake).

## Discussion

Our observation of a significant risk reduction in human colon cancer due to intake of selective COX-2 inhibitors is similar to that reported by Rahme et al. [9]. Standard daily dosages of celecoxib (200 mg) or rofecoxib (25 mg) were associated with a 69% reduction in colon cancer risk. Notably, comparator NSAIDs with non-selective COX-2 activity (325 mg aspirin or 200 mg ibuprofen) also produced significant risk reductions of similar magnitude. In contrast, the effect of low dose aspirin (81 mg) was only marginally significant and acetaminophen, an analgesic with little COX-2 activity, had no effect on the risk of colon cancer.

Selective COX-2 inhibitors (celecoxib and rofecoxib) were only recently approved for use in 1999, and rofecoxib (Vioxx) was withdrawn from the marketplace in 2004 [3-5]. Nevertheless, even in the short window of exposure to these compounds, the selective COX-2 inhibitors produced significant reductions in the risk of colon cancer, underscoring their strong potential for colon cancer chemoprevention.

In general, NSAIDs inhibit cyclooxygenase which is the key rate-limiting enzyme of prostaglandin biosynthesis [10-12]. Molecular studies show that the inducible COX-2 gene is over-expressed in human colon cancer and that genetic expression of COX-2 in cancer cells is correlated with mutagenesis, mitogenesis, angiogenesis, and deregulation of apoptosis [13-15]. Over the counter NSAIDs have consistently shown antitumor effects in animal models of carcinogenesis [16], and striking antitumor effects of the specific COX-2 inhibitor, celecoxib, have been observed against colon cancer [17]. Epidemiologic studies and randomized clinical trials provide convincing evidence that regular intake of aspirin and other NSAIDs not only inhibit the development of colon cancer *per se*, but also interrupt the evolution of preneoplastic lesions of the colonic mucosa [18]. Furthermore, recent randomized clinical trials of selective COX-2 inhibitors indicate that celecoxib suppresses the development of colon adenomas [19,20]. The current study coupled with existing clinical, preclinical and molecular evidence suggest that aberrant induction of COX-2 and up-regulation of the prostaglandin cascade play a significant role in colon carcinogenesis, and that blockade of this process has strong potential for intervention. It is noteworthy that NSAIDs also manifest anticancer effects by mechanisms other than COX-2 inhibition [21]. For example, celecoxib has been found to have multiple COX-independent anticancer effects including induction of apoptosis and inhibition of cell cycle progression, angiogenesis, and metastasis [21,22]. Thus, it will be important to exploit not only COX-2 blockade but also COX-independent molecular targets of these compounds [22].

Enthusiasm for the use of selective COX-2 blocking agents in the chemoprevention of colon cancer and other malignancies has been tempered by reports of adverse effects on the cardiovascular system leading to the recall of the popular anti-arthritic compound, rofecoxib (Vioxx) [23-25], and subsequently, the cardiovascular safety of all selective COX-2 inhibitors has come under scrutiny [5]. However, such studies involved supra-therapeutic dosages given over long periods of time without consideration of body size or individual differences in metabolism [26]. Nevertheless, not all studies reflect changes in cardiovascular risk with exposure to COX-2 inhibitors [27,28] and recently a large meta-analysis of existing randomized clinical trials found no risk increase at any dose level of celecoxib [29]. Further studies will be required to determine the appropriate dose, frequency of intake, duration, side effects and cost effectiveness of selective and nonselective COX-2 inhibitors in the chemoprevention of cancer.

## Conclusion

We observed a significant reduction in the risk of human colon cancer due to intake of both selective and nonselective COX-2 inhibitors. Chemopreventive effects against colon cancer were associated with recommended daily doses of celecoxib (median dose = 200 mg) or rofecoxib (median dose = 25 mg) for an average duration of 3.6 years. Notably, the regular intake of over the counter NSAIDs such as aspirin and ibuprofen produced risk reductions in colon cancer similar in magnitude to the selective COX-2 inhibitors.

## Competing interests

This research was supported in part by a grant from Pfizer, New York, NY, and grant P30 CA16058 from the National Cancer Institute, Bethesda, MD.

## Authors' contributions

REH designed and directed the study. JBD coordinated data collection and quality control, and assisted in the interpretation of results. GAA assisted in the analysis and interpretation of results. All authors read and approved the final manuscript.

## Pre-publication history

The pre-publication history for this paper can be accessed here:


